# Comparison of Two Methods for *In Vivo* Estimation of the Glenohumeral Joint Rotation Center (GH-JRC) of the Patients with Shoulder Hemiarthroplasty

**DOI:** 10.1371/journal.pone.0018488

**Published:** 2011-03-31

**Authors:** Ali Asadi Nikooyan, Frans C. T. van der Helm, Peter Westerhoff, Friedmar Graichen, Georg Bergmann, H. E. J. (Dirkjan) Veeger

**Affiliations:** 1 Department of Biomechanical Engineering, Delft University of Technology, Delft, The Netherlands; 2 Julius Wolff Institut, Charité, Universitätsmedizin, Berlin, Germany; 3 Research Institute MOVE, VU Amsterdam, Amsterdam, The Netherlands; University of Pittsburgh, United States of America

## Abstract

Determination of an accurate glenohumeral-joint rotation center (GH-JRC) from marker data is essential for kinematic and dynamic analysis of shoulder motions. Previous studies have focused on the evaluation of the different functional methods for the estimation of the GH-JRC for healthy subjects. The goal of this paper is to compare two widely used functional methods, namely the instantaneous helical axis (IHA) and symmetrical center of rotation (SCoRE) methods, for estimating the GH-JRC *in vivo* for patients with implanted shoulder hemiarthroplasty. The motion data of five patients were recorded while performing three different dynamic motions (circumduction, abduction, and forward flexion). The GH-JRC was determined using the CT-images of the subjects (geometric GH-JRC) and was also estimated using the two IHA and SCoRE methods. The rotation centers determined using the IHA and SCoRE methods were on average 1.47±0.62 cm and 2.07±0.55 cm away from geometric GH-JRC, respectively. The two methods differed significantly (two-tailed *p*-value from paired t-Test ∼0.02, post-hoc power ∼0.30). The SCoRE method showed a significant lower (two-tailed *p*-value from paired t-Test ∼0.03, post-hoc power ∼0.68) repeatability error calculated between the different trials of each motion and each subject and averaged across all measured subjects (0.62±0.10 cm for IHA vs. 0.43±0.12 cm for SCoRE). It is concluded that the SCoRE appeared to be a more repeatable method whereas the IHA method resulted in a more accurate estimation of the GH-JRC for patients with endoprostheses.

## Introduction

According to the International Society of Biomechanics (ISB) recommendation for the upper extremity [1], the glenohumeral joint rotation center (GH-JRC) is needed to define the local coordinate system and longitudinal axis of the humerus. The GH-JRC is impossible to palpate *in*-*vivo* and, thus, needs to be estimated.

A variety of methods have been developed for the estimation of the kinematic joint rotation centers of ball joints [2]. For estimation of the GH-JRC, various methods have been introduced and used such as regression models [3], [4], spherical-fit [5], instantaneous helical axis (IHA) [6], [7], [8], symmetrical center of rotation (SCoRE) [2], [9], bias compensated [10], and least-square methods [11], [12]. Nevertheless, there is disagreement about either “repeatability” or “accuracy” of those methods for approximation of the kinematic GH-JRC.

As for repeatability, Stokdijk *et al* [13] applied three methods, including a linear regression model, a spherical-fit, and the IHA method to calculate the GH-JRC *in-vivo*. They concluded that the sphere-fit and IHA methods gave almost identical results, but different to the regression method. They preferred the IHA over the spherical-fit due to its shorter calculation time. Monnet *et al* [9] used the SCoRE method for *in*-*vivo* estimation of the GH-JRC and compared it with the IHA method and concluded that SCoRE was a more repeatable method.

The studies who evaluated the accuracy of the different methods may be divided into the *in*-*vitro* and *in*-*vivo* studies:

The *in*-*vitro* studies have been carried out on cadavers. Veeger [6] compared the kinematic and geometric GH-JRC based on a cadaver study. He showed that the calculated GH-JRC using the IHA method was very close (≤2 mm) to the geometric center of rotation which was defined as the center of the sphere fitted to the glenoid surface with the radius of the humeral head [14].

In the *in*-*vivo* studies [3], [15], the geometric (anatomical) GH-JRC determined on the subject specific CT/MRI-images were used as the reference for evaluation of the accuracy of the functional methods for estimation of the kinematic GH-JRC. Campbell *et al* [3] used MRI images to evaluate a newly developed regression model. In the most comprehensive study [15], five different functional methods including IHA, SCoRE, bias compensated and two least square methods were compared based on the Euclidian distance between the kinematic GH-JRC and the geometrical GH-JRC pointed on the MRI images. Based on the results of [15], the SCoRE method approximated the geometrical GH-JRC more accurate than the IHA method. However, in contrast to the results of study by Monnet *et al* [9], the IHA method was the method which showed higher repeatability.

All the aforementioned *in*-*vivo* studies were carried out on healthy subjects. Nevertheless, based on our best knowledge, no functional method for *in*-*vivo* estimation of the GH-JRC has yet been evaluated for patients with endoprostheses for whom the displaced rotation centers may occur. In the current study we will focus on the two recently most debated methods i.e. the IHA and the SCoRE. The aim of this paper is to evaluate the repeatability as well as the accuracy of the IHA and SCoRE methods for *in*-*vivo* estimation of the kinematic GH-JRC for the patients who carry the shoulder hemi-endoprosthesis. The repeatability of each method will be accessed across different motion trials for each subject. To evaluate the accuracy, the geometric GH-JRC determined on the post-operative CT-scan images of the patients will be used as the reference of comparison.

## Methods

### 1. Ethics statement

The ethical committee of the Freie Universität Berlin and Charité-Universitätsmedizin Berlin gave permission for the clinical studies using the shoulder endoprosthesis and post-operative CT-scans. Before surgery, the patients were informed about the aims and procedures of all measurements after which they agreed by signing an informed consent to participate and having their images published.

### 2. Data recordings

#### 2.1. Subjects

Five patients with a shoulder hemi-arthroplasty (Table 1) participated in the measurements. The patients were operated due to the diagnosis of osteoarthritis without serious rotator cuff damage. The surgery approach was deltopectoral. The endoprostheses were spherical (for the implant head radii see Table 1).

**Table 1:** Detailed information for the measured subjects.

Subject	Sex	Age	Implant side	Post-surgery CT (months)	Post-surgery Measure (months)	Implant head radius (mm)
S1	female	73	Left	5	7	24.0
S2	male	64	Right	9	9	22.0
S3	male	69	Right	11	16	24.0
S4	male	74	Right	6	11	25.0
S5	female	83	Right	-	30	22.0

#### 2.2. CT-imaging

Before and after joint replacement, 3D CT-scans of the subjects' upper extremity were obtained using a 64-slice CT scanner (Toshiba Aquilion 64, TMSE, The Netherlands) with slice thickness of 0.5 mm. Subject S5 (Table 1) was the only exception for whom only the pre-operative CT data was obtained. The CT-imaging was carried out in Charité Department of Radiology CCM, Berlin. All CT scans were taken in spine position.

#### 2.3. Motion data collection

Motion recordings were performed at Research Institute MOVE, Free University Amsterdam. Measurements included calibration, static, and dynamic trials. In the calibration process, the spatial positions of the anatomical landmarks on the thorax, scapula, and upper arm in the global coordinate system were recorded. Each anatomical landmark was palpated two times and the mean value of the two measured points was selected as the position of the bony landmark. For motion recordings, the spatial positions of the marker clusters on bony segments, including thorax, scapula, and upper arm, were captured using four Optotrak (Northern Digital Inc., Canada, nominal accuracy 0.3 mm) camera bars with the sampling frequency of 50 Hz. Each marker cluster included three markers.

The dynamic tasks included circumduction, abduction-adduction, and forward flexion (arm elevation and return to the initial position). The speed of the movements on average across all subjects was about 0.17 Hz (one cycle every 6 s). The subjects were asked to perform the abduction and flexion tasks up to maximum possible arm elevation. However, the measured subjects showed relatively limited elevation capacity (105°±25°). During the calibration and static trials, a scapula-locator [16], [17] was used together with cluster markers on the acromion (scapula-sensor [18], [19]), for more accurate scapular motion tracking. Both methods showed almost the same joint angles (differences <4°). We, therefore, decided to use the acromion sensor to follow the scapular motion during dynamic trials where using the scapula-locator was hardly possible.

### 3. Geometric GH-JRC

A cross-platform image processing software, namely the Delft Visualisation and Image processing Development Environment (DeVIDE version 9.8., Delft, the Netherlands) [20], was used to process the post-operative CT-Scan images.

To calculate the accuracy of point positioning on the CT images in DeVIDE, a set of six anatomical landmarks (incisura jugularis on the thorax, angulus acromialis, trigonum spinae, and angulus inferior on the scapula, epicondyle medialis and lateralis on the humerus) were pointed on the CT images based on the information provided in the ISB standardization proposal [1]. In the next step, the software was reloaded and the same bony landmarks as the last step were re-pointed on the CT scan images. Finally, the differences between the corresponding landmarks in the two sessions were calculated and the maximum value across all subjects (0.621 mm) was defined as the accuracy.

The image processing was performed manually. The anatomical bony landmarks on scapula including Angulus Acromialis (AA), Angulus Inferior (AI), and Trigonum Spinae (TS) were located on the images. The ISB standardization proposal was used for definition of the anatomical bony landmarks as well as the local coordinate definitions.

Alternative image processing software namely Mimics (version 13.1, Materialise, Leuven, Belgium) was also used for positioning of the anatomical landmarks. Since Mimics is more user-friendly and segmentation is easier controllable, we wanted to be certain that the originally used method (DeVIDE) provided trustable data. So, this process might probably be called a check on the reliability of segmentation and landmark identification. The maximum differences between the results in the two software (DeVIDE and Mimics) across all subjects did not exceed 2 mm. The landmarks identified in DeVIDE were used for further processing.

The geometric GH-JRC was determined on the CT scan images by using the method proposed and used in [14], [21]. van der Helm *et al* [14] showed that the surfaces of the glenohumeral joint are two concentric spheres and defined the center of sphere fitted to the glenoid using a constant radius equal to the radius of the humeral head. This definition was also used to determine the geometric GH-JRC in references [4], [21]. The method by van der Helm *et al* is, however, slightly different from previous studies [3], [15] in which the geometric GH-JRC was considered to be the center of the sphere fitted to the congruent surface of the humeral head. If the glenoid and humerus surfaces are congruent and in close contact, there should be no difference between both methods.

**Figure 1:**
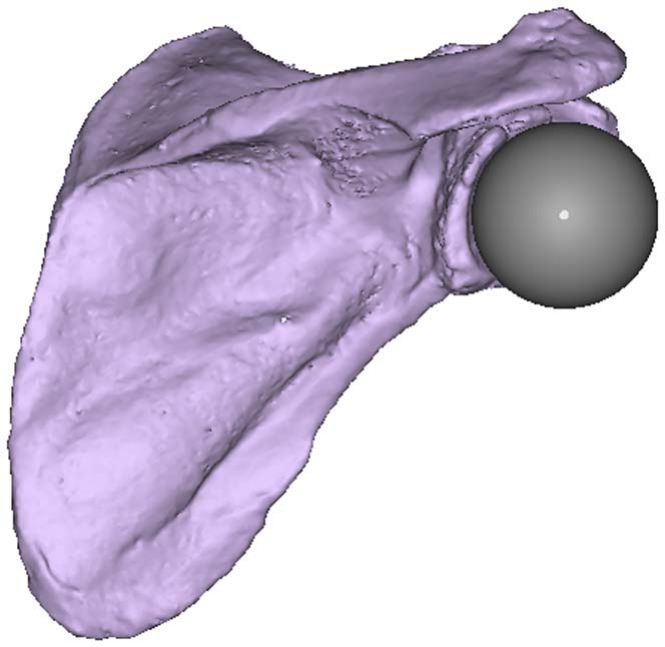
Visualization of the sphere fitted to the glenoid in Mimics software. For subject S4.

In order to find the radius of the humeral in previous studies [3], [15], [21], the positions of some points were determined on the caput humeri and subsequently a sphere was fitted to the data points using the least square method. However, in case of our patients, the radius of the humeral head will be equal to the radius of the implant head. Therefore, having the values of the implant head radius (Table 1), about 50 points on the glenoid surface (including the labrum) were determined on the segmented CT images of each subject. A sphere with the fixed radius of the implant head was fitted to the obtained data points on the glenoid surface by applying a least square criterion [6]. The center of the fitted sphere was defined to be the geometric GH-JRC. The fitted sphere was also visualized on the CT images in the Mimics software (Figure 1) to check the correctness of the mathematical calculations.

In contrast to subjects S1 to S4, for subject S5 the pre-operative CT data were used since post-operative images worked out to be unobtainable. For this subject, the rotation center was determined using the known geometry of the humeral head and the shape of the glenoid, assuming a tight contact between the two. As a check, we compared the segmented glenoid on the pre- and postoperative images for subject S1 to S4 and did not observe any changes in the shape of the glenoid and/or scapula.

### 4. Kinematic GH-JRC

#### 4.1. The IHA method

**Figure 2:**
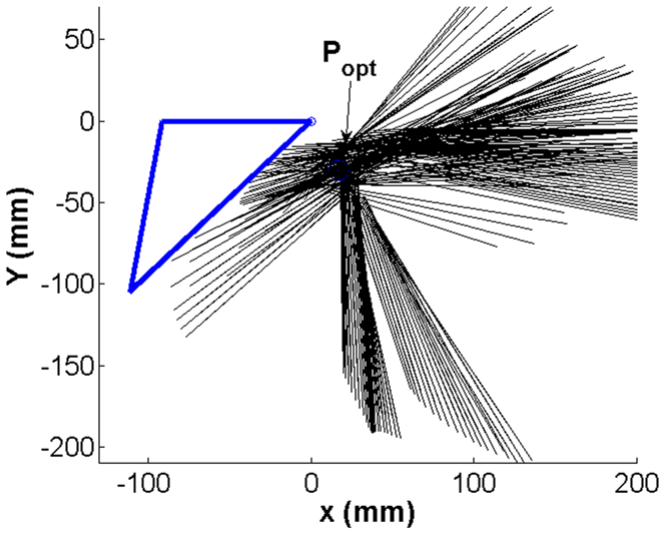
Typical example of the calculated instantaneous helical axes for Cir, Abd, and FE motions in the *xy*-plane. Selected axes are plotted for each motion dataset. P_opt_: the optimal pivot point (the kinematic GH-JRC calculated using the IHA- method).

In the IHA method (for details see references [7], [8], [22]), at each time frame of the data recording, the position vector (**p**) of an instantaneous helical axis (Figure 2) is calculated using the relative position vector (**s**) as well as the angular velocity vector (**ω**) of the markers on the upper arm with respect to the markers on the scapula as follows:
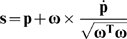
(1)Where **ω** is calculated from the rotation matrix (**R**) of the upper arm with respect to the scapula and its numerical derivative (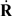) as follows:
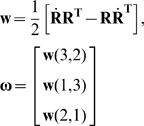
(2)The optimal pivot point (i.e. P_opt_, Figure 2) which is the closest point to all calculated helical axes is estimated by using the least squares optimization method developed by Woltring [22]. The estimated pivot point is defined as the kinematic joint rotation center.

#### 4.2. The SCoRE method

The SCoRE method (for details see references [2], [9]) is based on the assumption that the position of the joint rotation center should remain constant relative to the distal and proximal segments during performing a joint movement. As for the GH-joint, such assumption will mathematically result to the following linear least square problem:
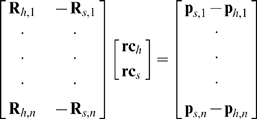
(3)Where:

**R** *_h_*_,*i*_ and **R** *_s_*_,*i*_ are, respectively, the rotation matrices of the upper arm (humerus) and scapula in the global coordinate system at time frame *i*.

**p** *_h_*_,*i*_ and **p** *_s_*_,*i*_ are, respectively, the position vector of the humerus and scapula in the global coordinate system at time frame *i*.

**rc** *_h_* and **rc** *_s_* are the position vector of the joint rotation center in the local coordinate system of the humerus and scapula, respectively.

#### 4.3. Estimation of the kinematic GH-JRC

The kinematic rotation center was calculated using both IHA and SCoRE methods. The position of the marker clusters on scapula and upper arm while performing dynamic trials were used. Similar to the previous studies who compared the IHA and SCoRE methods [9], [15] and in line with recommendations of Begon *et al* [23] for estimation of kinematic rotation center of the hip joint, three sets of kinematic data were used to find the joint rotation center as follows:

- Dataset 1 (Cir): one trial of arm circumduction motion
- Dataset 2 (FE/Abd): combination of one trial forward flexion (arm elevation and backing to the initial position) and one trial arm abduction/adduction
- Dataset 3 (FE/Abd/Cir): combination of one trial forward flexion, one trial abduction/adduction, and one trial circumduction

For each dataset, six trials were measured and used.

All kinematic data were filtered using a second order low-pass digital Butterworth filter with cutoff frequency of 3 Hz (∼18 times larger than the speed of movement). Due to the sensitivity of the IHA method to the angular velocity (ω, Eq. 2), only the angular velocities more than 10% of peak angular velocity (ω*_max_*, the highest norm angular velocity in the signal) were applied.

### 5. Repeatability of the methods

The repeatability of the methods was evaluated in the same way as in [9], [15] based on the repeatability error (i.e. *e*, Table 2). The location of the GH-JRC in the space (*x*, *y*, *z*) was calculated with the two methods. For each type of motion dataset (1, 2, or 3) and each subject, the repeatability error (*e*) was defined as follows:
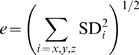
(4)Where SD*_x_* is the standard deviation of the estimated GH-JRC locations in the *x*-direction among all six trials in each dataset. The same definition applies to the *y* and *z* directions.

**Table 2:** The repeatability error (*e*) for the IHA and SCoRE methods.

	IHA			SCoRE		
*e* (cm)	Cir	FE/Abd	FE/Abd/Cir	Cir	FE/Abd	FE/Abd/Cir
**S1**	1.02	0.92	0.74	0.80	0.83	0.57
**S2**	0.81	0.77	0.51	0.57	0.44	0.29
**S3**	0.96	0.53	0.57	0.90	0.76	0.48
**S4**	0.78	0.84	0.55	0.53	0.56	0.47
**S5**	0.21	0.98	0.72	0.30	0.44	0.32
**mean**	0.76	0.81	0.62	0.62	0.61	0.43
**SD**	0.32	0.17	0.10	0.24	0.18	0.12

The lower repeatability error means more repeatability for a method.

### 6. Accuracy of the methods

The accuracy of each method was accessed by calculation of the Euclidian distance (i.e. *d*, Table 3) between the estimated and the geometric GH-JRC (see section 2.3), as was carried out by Lempereur *et al* [15]. To allow for a direct comparison between the estimated and geometric GH-JRC, they should be represented at the same coordinate system. Using the three scapular bony landmarks (AA, TS, and AI) on the CT-scan images and experimental data, the local coordinate system of the scapula was defined as the reference coordinate system. The direction of the scapular coordinate system axes was chosen similar to previous studies [9], [13] with the *x*-axis pointing to the right, the *y*-axis pointing upward, the *z*-axis pointing backward, and the origin at AA. The scapular coordinate system obtained from the *in*-*vivo* measurements was aligned to the one derived from the CT images using the optimization method described by Veldpaus *et al* [24]. The AA point was selected as the basis point for transformations between the two local coordinate systems. The kinematic GH-JRCs were then transferred to the aligned coordinate system.

**Table 3:** The 3D positions of the scapular anatomical landmarks as well as the kinematic and geometric GH-JRC.

		Anatomical Landmarks						Geometric	Kinematic GH-JRC					
		AA		TS		AI		GH-RC	IHA			SCoRE		
		CT	Kin.	CT	Kin.	CT	Kin.	CT	Cir	FE/Abd	FE/Abd/Cir	Cir	FE/Abd	FE/Abd/Cir
**S1**	***x***	0	0	−9.13	−11.24	−11.12	−11.57	1.31	1.35	0.69	0.66	1.18	0.48	0.72
	***y***	0	0	0	0.78	−10.48	−9.96	−2.83	−3.19	−2.72	−2.69	−2.76	−2.9	−2.85
	***z***	0	0	0	−0.25	0	0.19	−3.12	−3.98	−3.55	−3.49	−4.19	−5.38	−4.99
	***d***	-	0	-	2.26	-	0.71	-	0.93	0.76	0.76	1.08	2.41	1.96
**S2**	***x***	0	0	−11.77	−10.85	−12.80	−11.94	−0.45	0.66	0.55	0.54	0.02	−0.05	−0.04
	***y***	0	0	0	1.48	−10.45	−11.00	−2.89	−3.8	−3.56	−3.63	−4.14	−3.06	−3.61
	***z***	0	0	0	−0.25	0	0.19	−2.45	−2.14	−3.24	−2.64	−2.96	−4.28	−3.64
	***d***	-	0	-	1.76	-	1.04	-	1.47	1.44	1.25	1.43	1.88	1.45
**S3**	***x***	0	0	−12.48	−12.45	−12.35	−12.05	−0.95	0.06	−2.01	−1.9	−1.13	−1.28	−1.23
	***y***	0	0	0	0.32	−15.02	−14.67	−3.29	−5.57	−4.24	−4.39	−6.78	−4.7	−5.4
	***z***	0	0	0	0	0	0	−3.43	−3.33	−2.6	−2.81	−4.73	−3.95	−4.21
	***d***	-	0	-	0.32	-	0.46	-	2.5	1.64	1.58	3.73	1.54	2.26
**S4**	***x***	0	0	−11.96	−11.13	−13.16	−13.26	1.39	0.46	0.44	0.46	0.62	1.24	1.04
	***y***	0	0	0	0.47	−11.47	−12.64	−2.82	−3.55	−4.5	−3.69	−3.47	−4.75	−4.33
	***z***	0	0	0	0	0	0	−4.22	−3.82	−4.3	−3.84	−3.54	−3.26	−3.36
	***d***	-	0	-	0.95	-	1.17	-	1.25	1.93	1.33	1.21	2.16	1.77
**S5**	***x***	0	0	−10.58	−10.00	−11.69	−11.62	−0.50	−1.72	−1.69	−1.69	−1.91	−1.43	−1.53
	***y***	0	0	0	1.20	−10.85	−12.10	−2.54	−5.36	−3.77	−4.61	−6.64	−4.55	−4.97
	***z***	0	0	0	0	0	0	−2.83	−2.29	−2.32	−2.30	−3.18	−1.23	−1.62
	***d***	-	0	-	1.33	-	1.25	**-**	3.12	1.78	2.44	4.35	2.73	2.90
**mean**	***x***	0	0	−11.18	−11.13	−12.22	−12.09	0.16	0.16	−0.40	−0.39	−0.24	−0.21	−0.21
	***y***	0	0	0	0.85	−11.65	−12.07	−2.87	−4.29	−3.76	−3.80	−4.76	−3.99	−4.23
	***z***	0	0	0	−0.10	0	0.08	−3.21	−3.12	−3.32	−3.06	−3.72	−3.67	−3.65
	***d***	-	0	-	0.86	-	0.45	-	1.85(0.92)	1.51(0.46)	1.47(0.62)	2.36(1.55)	2.14(0.46)	2.07(0.55)

The AA point was used as the basis for aligning the measured and CT-based landmarks.

All values are in cm.

Kin.: kinematic.

*d*: the Euclidian distance between the kinematic and the CT-based GH-JRC.

### 7. Statistical analysis

Two-tailed paired Student's t-Test was used for statistical analysis. The threshold for statistical significance was considered as 0.05. Post-hoc statistical power analysis for two-tailed Student's t-Test was carried out in order to evaluate the power of test with low number of subjects (*n* = 5).

## Results

### 1. Repeatability of the methods

Comparison of the repeatability error (*e*) for the three datasets in each method and for all subjects showed that the minimum value for the average error was 0.62 and 0.43 cm for the IHA and SCoRE methods respectively (Table 2).

### 2. Accuracy of the methods

Differences up to 2.26 cm (TS point for S1, Table 3) appeared between the calibration positions of the *in*-*vivo* measured and CT-pointed bony landmarks.

**Figure 3:**
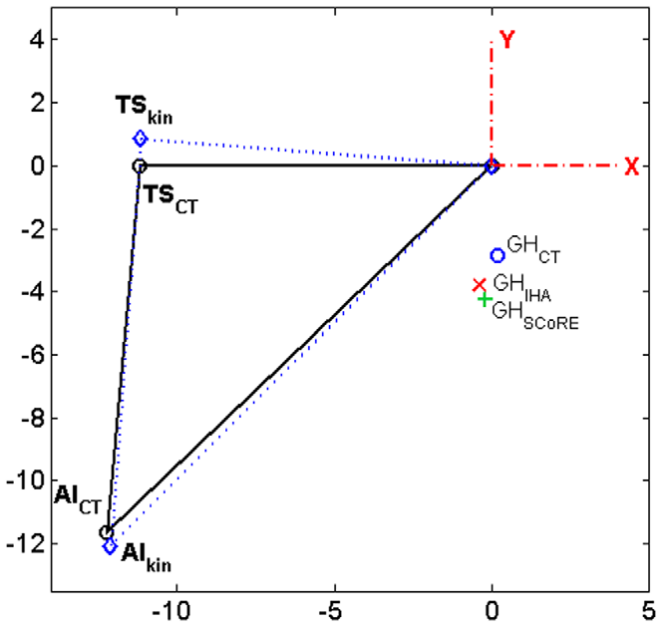
The kinematic and geometric GH-JRC as well as the scapular anatomical landmarks in the *xy*-plane. The mean values of the four subjects in Table 3 are used. The AA-point was used to align the kinematic (GH_IHA_ and GH_SCoRE_) and CT-based (GH_CT_) coordinate systems. The axes are in cm.

The estimated kinematic GH-JRC for the IHA was on average 1.47 cm away from the geometric GH-JRC. For the SCoRE value this amounted to 2.07 cm (Table 3, Figure 3).

The closest GH-JRC predicted by IHA method had a distance of about 0.76 cm from the geometric GH-JRC while the best point estimated by the SCoRE method differed about 1.08 cm from the CT-estimated JRC, both related to S1 (Table 3).

The distance between the kinematic GH-JRCs calculated using the IHA and SCoRE methods for motion datasets Cir, FE/Abd, and FE/Abd/Cir was, respectively, 0.83 cm, 0.50 cm, and 0.78 cm. The same quantities were reported to be, respectively, 1.41 cm, 0.72 cm, and 0.46 cm in reference [9]. The mean difference between the two methods in the study by Lempereur *et al* [15] was 0.48 cm.

### 3. Statistics

The difference between the IHA and SCoRE method for the distance to the geometrical GH-JRC (*d*) was significant (two-tailed *p*-value ∼0.02, post-hoc power ∼0.30, Table 4) for Dataset 3 (FE/Abd/Cir).

**Table 4:** The results of the paired t-Test and post-hoc power analysis.

	2-tailed *p*-value		Post-hoc power	
	*d*	*e*	*d*	*e*
**Cir**	0.16	0.11	0.08	0.10
**FE/Abd**	0.11	0.19	0.48	0.36
**FE/Abd/Cir**	0.02*	0.03*	0.30	0.68

*e*: the repeatability error.

*d*: the Euclidian distance between the kinematic and geometric GH-JRC.

*: significant difference (*p*<0.05).

## Discussion

This study compared two methods (SCoRE and IHA) for estimation of the GH-JRC for subjects with the shoulder hemi-arthroplastic endoprosthesis based on the distance to the geometric GH-JRC obtained from the subject-specific post-operative CT scans. The results for the IHA and SCoRE method were not the same: the IHA results were significantly closer to the rotation center than the SCoRE results. The difference between the estimated IHA and SCoRE centers was comparable to the similar studies on healthy adults [9], [15].

The comparison between the functional methods for estimation of the GH-JRC may be carried out based on either “repeatability” or “accuracy”.

As for repeatability, Monnet *et al* [9], found a lower repeatability error when using the SCoRE method (0.30 cm) as compared to the IHA method (0.43 cm), as we found in the current study, while Lempereur *et al* [15] reported slightly higher repeatability error for the SCoRE method (4.36 cm vs. 4.11 cm for the IHA method). This means that there is not yet consensus about which method is more repeatable, even for the studies on healthy subjects. However, one should note that the results of the study by Monnet *et al* [9] are statistically more reliable than the study by Lempereur *et al* [15] due to its larger number of participants (10 vs 4). The difference between the results of the different studies may be related to the fixed error sources:

Both the SCoRE and the IHA methods start from the assumption that there is a GH-JRC with only three rotational degrees of freedom. This definition implies that translations within the joint are minimal. This assumption could potentially be a source of fixed errors. According to Graichen *et al* [25] this is a valid assumption, since their MRI study of glenohumeral motions indicated mean glenohumeral translations during humeral elevation up to 1.2 mm. In our study translations were quite small and did not show a systematically changing position. Should, however, translations occur within the joint, this position would change with joint angle. In cases of a compromised joint in which more random translations are occurring, both positions and directions of the axes would change randomly. The fact that IHA method results can be interpreted as indication for the validity of the 3 DOF assumption, can be seen as a strong point of this particular method, which is in fact the exact opposite of the argument used by Monnet *et al* [9] in their choice of the SCoRE over the IHA method.

Another source of fixed errors could be the assumption that there is a fixed relationship between the bony landmarks and the glenoid. Although the study by Meskers *et al* [4] has indicated that such a relationship exists and the assumption is therefore valid, it is, however, quite unlikely that there would be no interindividual variation at all.

Regarding the accuracy, the reference point (the geometric GH-JRC) used for evaluating the accuracy of the two methods was similar in the current study (on patients) and the study by Lempereur *et al* [15] (on healthy subjects). However, the results of the two studies are not identical. The difference between the studies may be due to the differences between the subjects (healthy vs patients with implants), which is not very likely, or related the random error sources. The potential sources for random errors could be the sampling errors of the motion capture system, the tissue artifact effects on motion of the technical markers [26], digitization errors of the flock of bird systems [17], treatment of the *in*-*vivo* measured data (e.g. filtering frequency, type of filter, etc.), errors in manual CT/MRI image processing, and inter-coordination transformation (from *in*-*vivo* measured to CT/MRI system or vice versa) errors (e.g. using the alternate examining basis point).

Accurate estimation of the GH-JRC is demanded for various applications. As a kinematic application, it is needed to define the local coordinate system of the upper arm as was stated in the ISB standardization protocol for the upper extremity [1]. A more important application would be in subject-specific modeling. According to the recent studies [27], it is now clear that to estimate reliable (muscle and joint reaction) forces, the musculoskeletal model should be scaled to subject-specific characteristics. Inaccuracies in estimation of the GH-JRC may cause considerable errors in calculation of some critical parameters (e.g. moment arms, origins and insertions of the muscles crossing the glenohumeral joint) in the scaled model.

The ISB standardization protocol recommends the IHA method for estimating the GH-JRC *in-vivo* in case of patients with shoulder implantation for whom the displaced rotation centers may occur. Assuming the geometric GH-JRC derived from the post-operative CT-data to be our reference, the IHA showed a significantly closer approximation for the most generalized combination of shoulder movements. We conclude that the IHA method can be recommended for estimation of GH-JRC for patients carrying shoulder implants.

Finally, we conclude that the SCoRE appears to be a more repeatable method whereas the IHA method resulted in a more accurate estimation of the GH-JRC for patients with endoprostheses.

## References

[1] Wu G, van der Helm FCT, Veeger HEJ, Makhsous M, Van Roy P (2005). ISB recommendation on definitions of joint coordinate systems of various joints for the reporting of human joint motion-Part II: shoulder, elbow, wrist and hand.. Journal of Biomechanics.

[2] Ehrig RM, Taylor WR, Duda GN, Heller MO (2006). A survey of formal methods for determining the centre of rotation of ball joints.. Journal of Biomechanics.

[3] Campbell AC, Lloyd DG, Alderson JA, Elliott BC (2009). MRI development and validation of two new predictive methods of glenohumeral joint centre location identification and comparison with established techniques.. Journal of Biomechanics.

[4] Meskers CGM, van der Helm FCT, Rozendaal LA, Rozing PM (1998a). In vivo estimation of the glenohumeral joint rotation center from scapular bony landmarks by linear regression.. Journal of Biomechanics.

[5] Halvorsen K, Lesser M, Lundberg A (1999). A new method for estimating the axis of rotation and the center of rotation.. Journal of Biomechanics.

[6] Veeger HEJ (2000). The position of the rotation center of the glenohumeral joint.. Journal of Biomechanics.

[7] Woltring HJ, Huiskes R, de Lange A, Veldpaus FE (1985). Finite centroid and helical axis estimation from noisy landmark measurements in the study of human joint kinematics.. Journal of Biomechanics.

[8] Woltring HJ, Long K, Osterbauer PJ, Fuhr AW (1994). Instantaneous helical axis estimation from 3-D video data in neck kinematics for whiplash diagnostics.. Journal of Biomechanics.

[9] Monnet T, Desailly E, Begon M, Vallee C, Lacouture P (2007). Comparison of the SCoRE and HA methods for locating in vivo the glenohumeral joint centre.. Journal of Biomechanics.

[10] Halvorsen K (2003). Bias compensated least squares estimate of the center of rotation.. Journal of Biomechanics.

[11] Chang LY, Pollard NS (2007). Constrained least-squares optimization for robust estimation of center of rotation.. Journal of Biomechanics.

[12] Gamage SSHU, Lasenby J (2002). New least squares solutions for estimating the average centre of rotation and the axis of rotation.. Journal of Biomechanics.

[13] Stokdijk M, Nagels J, Rozing PM (2000). The glenohumeral joint rotation centre in vivo.. Journal of Biomechanics.

[14] van der Helm FCT, Pronk GM, Veeger HEJ, van der Woude LHV (1989). The rotation center of the glenohumeral joint.. Journal of Biomechanics.

[15] Lempereur M, Leboeuf F, Brochard S, Rousset J, Burdin V (2010). In vivo estimation of the glenohumeral joint centre by functional methods: Accuracy and repeatability assessment.. Journal of Biomechanics.

[16] Barnett ND, Duncan RD, Johnson GR (1999). The measurement of three dimensional scapulohumeral kinematics - a study of reliability.. Clinical Biomechanics.

[17] Meskers CGM, Vermeulen HM, de Groot JH, van der Helm FCT, Rozing PM (1998b). 3D shoulder position measurements using a six-degree-of-freedom electromagnetic tracking device.. Clinical Biomechanics.

[18] Karduna AR, McClure PW, Michener LA, Sennett B (2001). Dynamic measurements of three-dimensional scapular kinematics: A validation study.. Journal of Biomechanical Engineering-Transactions of the Asme.

[19] van Andel CJ, Wolterbeek N, Doorenbosch CAM, Veeger D, Harlaar J (2008). Complete 3D kinematics of upper extremity functional tasks.. Gait & Posture.

[20] Botha CP, Post FH, Hauser H, Strassburger S, Theisel H (2008). Hybrid scheduling in the DeVIDE dataflow visualisation environment.. Simulation and Visualization.

[21] van der Helm FCT, Veeger HEJ, Pronk GM, Van der Woude LHV, Rozendal RH (1992). Geometry parameters for musculoskeletal modelling of the shoulder system.. Journal of Biomechanics.

[22] Woltring HJ, Capozzo A, Berme P (1990). Biomechanics of Human Movement, Applications in Rehabilitation, Sport and Ergonomics;.

[23] Begon M, Monnet T, Lacouture P (2007). Effects of movement for estimating the hip joint centre.. Gait & Posture.

[24] Veldpaus FE, Woltring HJ, Dortmans LJMG (1988). A least-squares algorithm for the equiform transformation from spatial marker co-ordinates.. Journal of Biomechanics.

[25] Graichen H, Stammberger T, Bonel H, Karl-Hans E, Reiser M (2000). Glenohumeral translation during active and passive elevation of the shoulder – a 3D open-MRI study.. Journal of Biomechanics.

[26] Cereatti A, Donati M, Camomilla V, Margheritini F, Cappozzo A (2009). Hip joint centre location: An ex vivo study.. Journal of Biomechanics.

[27] Nikooyan AA, Veeger HEJ, Westerhoff P, Graichen F, Bergmann G (2010). Validation of the Delft Shoulder and Elbow Model using in-vivo glenohumeral joint contact forces.. Journal of Biomechanics.

